# GHOSTM: A GPU-Accelerated Homology Search Tool for Metagenomics

**DOI:** 10.1371/journal.pone.0036060

**Published:** 2012-05-04

**Authors:** Shuji Suzuki, Takashi Ishida, Ken Kurokawa, Yutaka Akiyama

**Affiliations:** 1 Graduate School of Information Science and Engineering, Tokyo Institute of Technology, Meguro-ku, Tokyo, Japan; 2 Graduate School of Bioscience and Biotechnology, Tokyo Institute of Technology, Yokohama-shi, Kanagawa, Japan; Aberystwyth University, United Kingdom

## Abstract

**Background:**

A large number of sensitive homology searches are required for mapping DNA sequence fragments to known protein sequences in public and private databases during metagenomic analysis. BLAST is currently used for this purpose, but its calculation speed is insufficient, especially for analyzing the large quantities of sequence data obtained from a next-generation sequencer. However, faster search tools, such as BLAT, do not have sufficient search sensitivity for metagenomic analysis. Thus, a sensitive and efficient homology search tool is in high demand for this type of analysis.

**Methodology/Principal Findings:**

We developed a new, highly efficient homology search algorithm suitable for graphics processing unit (GPU) calculations that was implemented as a GPU system that we called GHOSTM. The system first searches for candidate alignment positions for a sequence from the database using pre-calculated indexes and then calculates local alignments around the candidate positions before calculating alignment scores. We implemented both of these processes on GPUs. The system achieved calculation speeds that were 130 and 407 times faster than BLAST with 1 GPU and 4 GPUs, respectively. The system also showed higher search sensitivity and had a calculation speed that was 4 and 15 times faster than BLAT with 1 GPU and 4 GPUs.

**Conclusions:**

We developed a GPU-optimized algorithm to perform sensitive sequence homology searches and implemented the system as GHOSTM. Currently, sequencing technology continues to improve, and sequencers are increasingly producing larger and larger quantities of data. This explosion of sequence data makes computational analysis with contemporary tools more difficult. We developed GHOSTM, which is a cost-efficient tool, and offer this tool as a potential solution to this problem.

## Introduction

Metagenomics, which is the study of the genomes of uncultured microbes obtained directly from microbial communities in their natural habitats, has recently become more popular because of the rapid improvement in sequencing technologies. For example, a current Illumina/Solexa system can produce billions of base pairs (bp) of data on a single run of the machine, and the throughput of the system is approximately 1,000 times higher than that of previous sequencers [1]. However, current sequencers only produce information in short fragments, whose lengths range between 50 and 700 bp. Only a simple mapping process is required for single-organism genomics to identify the location of each DNA sequence fragment if the reference genome has already been obtained. For this purpose, many efficient short-read mapping programs, such as BWA [2], [3], Bowtie [4], and RMAP [5], have been developed.

However, in metagenomic analysis, the DNA sequence fragments obtained from environmental samples frequently include DNA sequences from many different species, and closely related reference genome sequences are often unavailable. Thus, more sensitive approaches are required for the identification of novel genes. In the typical metagenomic analyses, sequenced DNA fragments are translated into protein coding sequences and then further assigned to protein families, such as COG [6], [7] and Pfam [8]. The BLASTX [9], [10] program has been used for such binning and classification because it can identify homologues that do not have high nucleotide sequence identity, but once these sequences are translated, the homolog can be found in a distantly related member of a protein family [11], [12]. The BLAST algorithm is sufficiently sensitive for searching protein families and is much faster than the classical dynamic programming method used in SSEARCH [13]. However, its performance is insufficient for analyzing the large quantities of data produced by a next-generation sequencer. In practice, approximately 960 CPU days were needed for querying 20 million short reads against the KEGG database [14]–[16] using BLAST.

There are several faster homology search tools than BLAST. For example, BLAT [17] is one of the fastest homology search tools, and it is approximately 50 times faster than BLAST. However, the search sensitivity of BLAT is much lower than that of BLAST and is insufficient for identifying protein families. Thus, there is currently a strong demand for much faster tools for conducting sensitive sequence homology searches in metagenomic analyses.

**Table 1:** Computation time for 100 thousand reads.

Program	#GPUs	Time (sec.)	Acceleration ratio
GHOSTM	1	2,855	129.5
GHOSTM	4	909	406.7
BLAT		9,898	37.3
BLASTX (1 thread)		369,678	1.0
BLASTX (4 threads)		102,255	3.6

The first, second, third, and fourth columns show the name of each program, the number of GPUs used for the calculation, the computation time, and the acceleration in processing speed relative to BLAST using 1 thread, respectively.

Graphics processing units (GPUs) are architectures that were originally designed for graphics applications. However, new-generation GPUs have been transformed into powerful co-processors for general purpose computing, and their computational power supersedes that of CPUs. For example, the peak performance of a GPU, such as the Tesla C1060, is approximately 1 TFLOPS. This speed is more than 10-fold faster than the most recent CPUs. GPUs have already been used for several bioinformatics applications, such as CUDASW++ [18], [19] and GPU-HMMER [20]. These applications have successfully achieved more than a 5-fold increase in acceleration compared to their CPU-based counterparts. Using GPUs, the BLASTP program was also accelerated to create new applications, known as GPU-BLAST [21] and CUDA-BLASTP [22]. BLASTP performs protein versus protein sequence searches, whereas BLASTX conducts a translated DNA sequence search against a protein database with automatic translation of the query sequence into all six of the possible reading frames. However, the calculation speed of CUDA-BLASTP was only approximately 10 times faster than BLAST on the CPU platform, and GPU-BLAST was only approximately 3 times faster. The small increase in speed was likely related to the BLAST search algorithm being complicated and inefficient when implemented on GPUs. Therefore, a new and efficient search algorithm optimized for GPU calculations is required.

**Table 2:** Computation time for approximately 6.8 million reads.

Program	#GPUs	Time (sec.)	Acceleration ratio
GHOSTM	1	166,740	4.2
GHOSTM	4	47,995	14.6
BLAT		699,300	1.0

The first, second, third, and fourth columns show the name of each program, the number of GPUs used for the calculation, the computation time, and the fold increase in the acceleration in the processing speed relative to BLAT, respectively.

Here, we developed a new and efficient homology search algorithm suitable for GPU calculation and implemented the system on GPUs. The system accepts a large number of short DNA fragment sequences produced by a next-generation sequencer as the input and, like the BLASTX program, performs DNA sequence homology searches against a protein sequence database. We used NVIDIA CUDA to implement the GPU computing. The search system, which we named GHOSTM (GPU-accelerated HOmology Search Tool for Metagenomics), demonstrated a calculation speed that was 130 times faster with one GPU than BLAST on a CPU. This system should enable researchers to analyze large amounts of metagenomic data from next-generation sequencers, even with a small-scale workstation.

**Figure 1:**
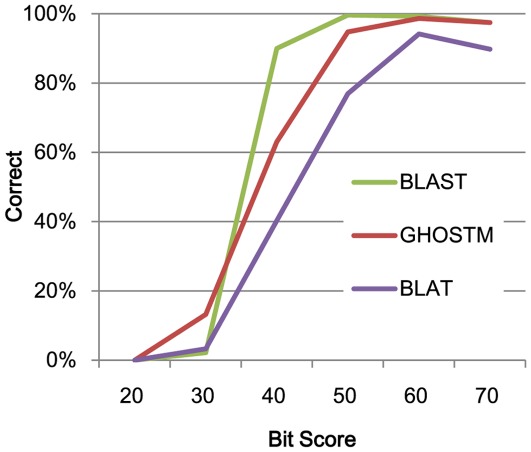
Search accuracy. The vertical axis shows the percentage of results for each method that corresponds to the correct answers. The horizontal axis shows the bit scores of the alignments.

## Results

### Datasets and Conditions

To evaluate the performance of GHOSTM, we compared its search accuracy and computation time with the NCBI BLAST+ package (version 2.2.25) and BLAT (standalone package, version 34). We used protein sequences obtained from KEGG Genes (“genes.pep”) as of November 2010 as the search target database. The number of sequences in the database was approximately 4.2 million, and the total length of these sequences was approximately 2.0 billion amino acids. We used DNA sequence reads obtained from a polluted soil metagenome study with Illumina/Solexa sequencing as the DNA query sequences. We used approximately 6.8 million high-quality reads selected from approximately 20 million reads that were obtained from the Illumina/Solexa sequence run. We selected reads that had a quality score greater than 15 (Q15 or over) over a continuous region of more than 60 bp. Thus, the lengths of the reads ranged from 60 to 75 bp. For all of the evaluations, we used the BLOSUM62 matrix as the substitution score matrix and performed all of the tests on a workstation with two dual core CPUs (3.2-GHz Dual-Core AMD Opteron 2224 SE) and a GPU server (1.44-GHz Tesla S1070), which included 4 GPUs.

**Figure 2:**
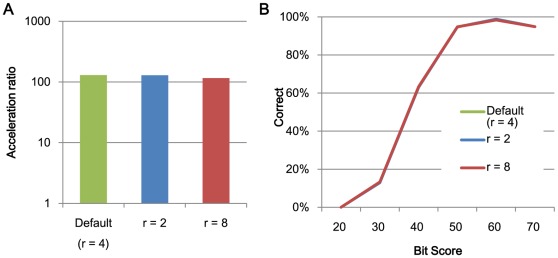
The relationships between search speed and accuracy and the search region size r. (A) The acceleration in processing speed relative to BLAST using 1 thread and (B) search accuracy.

**Figure 3:**
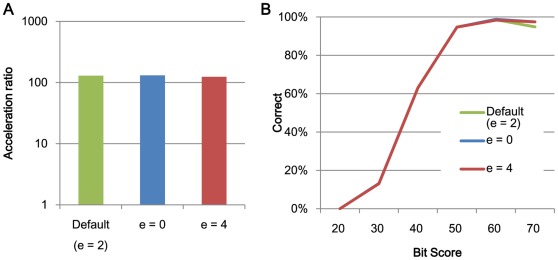
The relationships between search speed and accuracy and the extension size e. (A) The acceleration of processing speed relative to BLAST using 1 thread and (B) search accuracy.

**Figure 4:**
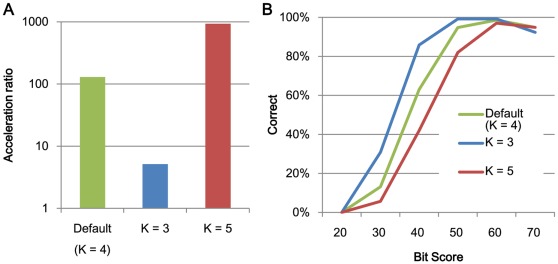
The relationships between search speed and accuracy and the length of search seeds K. (A) The acceleration of processing speed relative to BLAST using 1 thread and (B) search accuracy.

**Figure 5:**
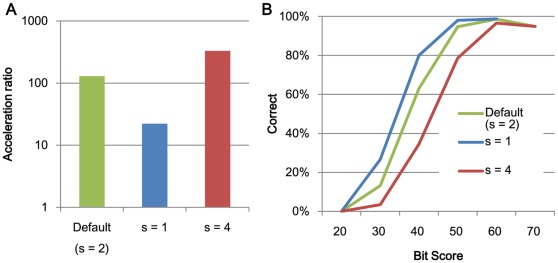
The relationships between search speed and accuracy and the character skips s. (A) The acceleration of processing speed relative to BLAST using 1 thread and (B) search accuracy.

**Figure 6:**
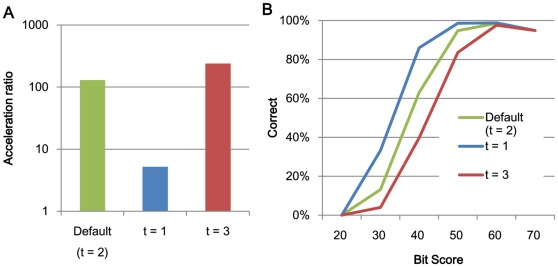
The relationships between search speed and accuracy and the number of required matches t. (A) The acceleration of processing speed relative to BLAST using 1 thread and (B) search accuracy.

### Evaluation of Computation Time

We ran GHOSTM, BLAST, and BLAT to measure their computation times. For comparing BLAT with GHOSTM, we used all of the 6.8 million reads as query sequences. However, we used only 100 thousand randomly selected reads as query sequences for comparing GHOSTM with BLAST because the calculation cost of BLAST is too excessive to perform millions of reads. As previously described, the queries were DNA reads, and the database was composed of protein sequences; thus, we executed the BLASTX program with the command line options ‘-outfmt 6 -seg no’, which instructed the program to output in tabular format. We did not use the SEG filter [23] because BLAST sometimes fails to find significant hits with this filtering for short queries. We tested BLASTX with 1 thread and 4 threads. The BLAT program does not include a function to translate DNA reads to protein sequences. Therefore, we translated the DNA reads into protein sequences based on the standard codon table. We executed the BLAT program with the command line option ‘–q = prot –t = prot –out = blast8,’ which instructed the program to use protein queries as well as a protein database and to output data in the BLAST tabular format. The BLAT program does not support a multi-core processor. Thus, we executed the BLAT with only 1 thread. For GHOSTM, we used the command line options ‘db -k 4 –l 128’ for constructing database indexes: the length of the search seeds was *K = 4*, and the size of a database chunk was 128 Mbp. Using these parameters, GHOSTM generated 16 database chunks for the KEGG Genes database. The command line options ‘aln –l 128 -s 2 -r 4 -e 2 -t 2’ were used for the search process, with character skips at *s =  2*, search region size at *r = 4*, extension size at *e = 2*, and the number of required matches at *t = 2*. We determined these parameters based on the balance between the prediction accuracy and computational time. The performance of GHOSTM with other parameters is discussed in the following section.

Table 1 shows the computational times for BLAST, BLAT, and GHOSTM for 100 thousand reads. The GHOSTM program achieved a calculation speed 129.5 and 35.8 times faster than the BLAST program using 1 thread and 4 threads, respectively. Moreover, GHOSTM was approximately 3.4 times faster than BLAT. In addition, GHOSTM implemented on a system with 4 GPUs showed a processing acceleration that was 406.7 and 112.5 times faster than the computational speed of BLAST using 1 thread and 4 threads, respectively. Thus, GHOSTM implemented on a system with 4 GPUs showed an acceleration that was approximately 3.1 times greater than the speed achieved using a single GPU.

**Table 3:** Computation time and acceleration of GHOSTM on a 1 GPU system relative to BLASTX for different query numbers.

#queries	GHOSTX (sec.)	BLASTX (sec.)	Acceleration ratio
1,000	213	4,180	19.6
10,000	422	37,167	88.0
100,000	2,855	369,678	129.5

Table 2 shows the computational times required for BLAT and GHOSTM to analyze the 6.8 million reads. The GHOSTM program was 4.2 times faster than the BLAT program. Moreover, GHOSTM implemented on a system with 4 GPUs showed a processing acceleration that was 14.6 times faster than BLAT. GHOSTM on a 4 GPU system was 3.5 times faster than the 1 GPU system for the 6.8 million reads, while the increase in speed with 4 GPUs was approximately 3.1 for the 100,000 reads.

**Figure 7:**
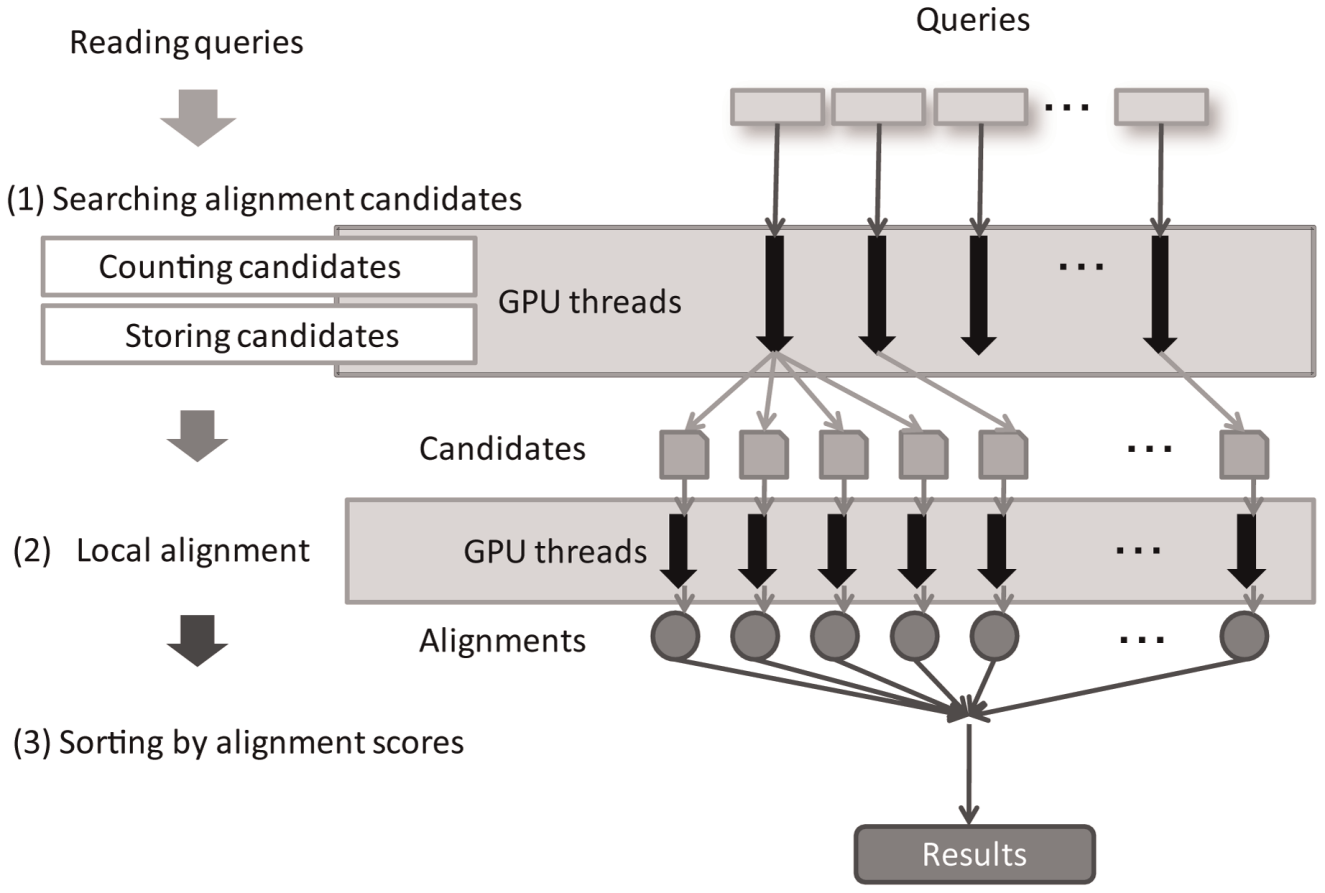
Data flow and processing within GHOSTM.

### Evaluation of Search Accuracy

To evaluate the search accuracy, we used the search results obtained with the Smith-Waterman local alignment method implemented in SSEARCH, and these results were assumed to be the correct answers. We analyzed the performance of a particular method in terms of the fraction of its results that corresponded to the correct answers obtained by SSEARCH.

For this analysis, we used only 10 thousand randomly selected reads because the calculation cost of the Smith-Waterman local alignment by SSEARCH was excessive. We translated the DNA reads into protein sequences in the same manner used for the evaluation of the computation time with BLAT because SSEARCH does not have a translation function. For these protein sequences, we executed the BLASTP program with the command line options ‘-outfmt 6 -seg no -comp_based_stats 0’. We did not use composition-based statistics [24] because this method was not employed in the default configuration of BLASTX. We also did not use the SEG filter. For GHOSTM and BLAT, we used the same command line options that were used for the evaluation of the computation time.

**Figure 8:**
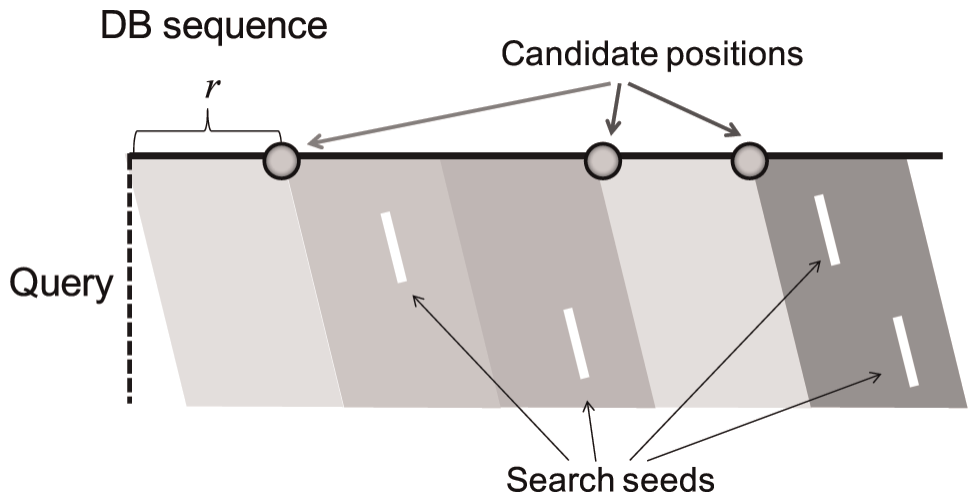
Search for candidate alignments.

Figure 1 shows the evaluation of the results of the search accuracy. The search accuracy of GHOSTM was clearly higher than BLAT. However, the accuracy of GHOSTM was lower than BLAST, especially for those hits whose scores were below 40. However, low-scoring hits (e.g., <50) are generally not used in practice because such hits can occur by chance. With the exception of the low-score hits, GHOSTM successfully identified more than 90% of the hits identified by SSEARCH. This result suggests that GHOSTM is sufficiently accurate for general usage.

**Figure 9:**
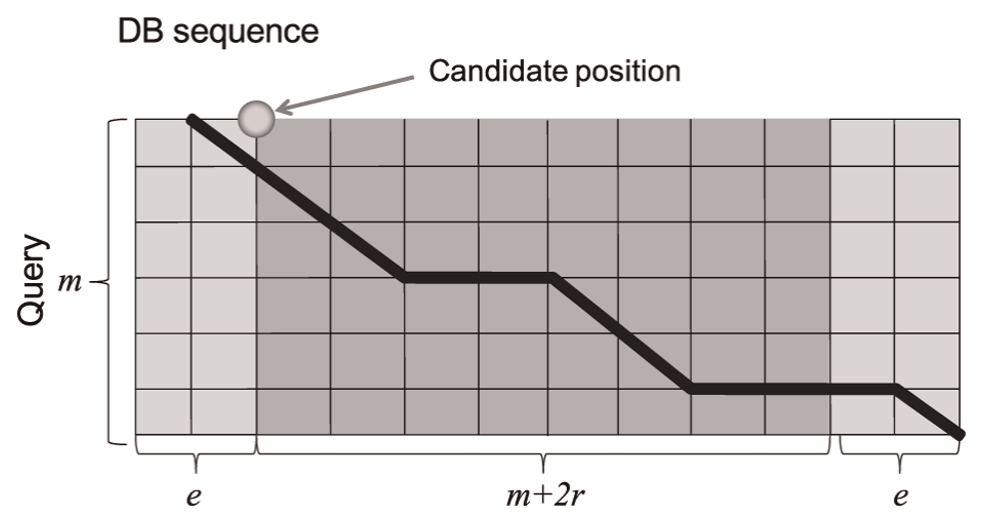
Calculation of an alignment in the region around a candidate position.

### Relationships between Search Parameters and their Accuracy and Computation Time

To determine the relationships between search parameters and their accuracy and computation time, we executed GHOSTM by changing one of its parameters from default to different values and measured the computation time and search accuracy. To evaluate the search accuracy and computation time, we used the same method used for comparing BLAST, BLAT and GHOSTM. We tested the following parameters and compared their computation times: *K = 3*, *4*, and *5*; *s = 2*, *3*, and *4*; *r = 2*, *4*, and *8*; *e = 0*, *2*, and *4*; and *t = 1*, *2*, and *3*.

Figure 2 and Figure 3 shows the acceleration in processing speed relative to GHOSTM with default parameters for different search regions size *r* and extension size *e*. As shown in the figure, search region size and extension size do not significantly change the search accuracy and computation time. However, other parameters, including the length of the search seed *K*, character skips *s,* and the number of required matches *t*, significantly change the performance. Using *K = 5*, *s = 4*, or *t = 3*, the acceleration of BLAST increases to 931.2, 329.5, and 239.6, respectively (Figures 4A, 5A, 6A). However, the search accuracies decrease to levels similar to BLAT (Figures 4B, 5B, 6B). With these parameters, GHOSTM often fails to find search seeds, including significant hits, which causes this low search accuracy. We believe that these search accuracies are insufficient for metagenomic analysis; thus, we did not use these settings as default settings. GHOSTM with *K = 3*, *s = 1*, or *t = 1* shows good search accuracy that is comparable with BLAST (Figures 4B, 5B, 6B.). However, the calculation speed is slower, and the acceleration of BLAST with 1 thread is 5.2, 22.2, and 5.2, respectively (Figures 4A, 5A, 6A). These accelerations are smaller than BLAT; thus, we did not use these parameters as default settings.

## Discussion

GHOSTM clearly outperformed BLAST in reducing the computation time for conducting homology searches. The reason for the acceleration in processing time was that the system simultaneously processed multiple queries on different GPU cores (the Tesla S1070 has 240 cores per GPU). Importantly, the GPU system requires a sufficient number of queries, and in fact, when using only one query sequence, the calculation of GHOSTM becomes much slower than BLAST. Table 3 shows the relationship between the number of query sequences and the acceleration in processing time. This result explains why GHOSTM on a system with 4 GPUs achieved a calculation speed that was only 3.1 times faster than GHOSTM on a system with 1 GPU for the small query set. However, the calculation speed of GHOSTM on a 4 GPU system was approximately 3.5 times faster than the speed obtained on the 1 GPU system when the number of queries was sufficient, as shown for 6.8 million reads. Thus, we suggest that the acceleration of GHOSTM will increase almost linearly as a function of the number of GPUs in practical situations in metagenomic analysis projects comprising hundreds of millions of reads.

In addition to the number of queries, GHOSTM had another restriction because it assumed that the length of all of the queries was approximately the same. For calculating the local alignment of each query, GHOSTM takes a GPU memory allocation plan according to the length of the longest query. Once GPU memory is allocated according to the maximum memory consumption case at first, GHOSTM can reuse the allocated space until the end of calculation, with avoiding overhead of GPU memory re-allocation. Thus, if the lengths of the queries were markedly different, GHOSTM required too much memory, which decreased the number of queries that GHOSTM could process concurrently. However, the number of reads from next-generation sequencers is large, and the lengths of the reads are approximately the same. Therefore, these two restrictions are generally satisfied, and we predict that they will have little impact on the calculation speed of GHOSTM.

### Methods

Our homology search tool was mainly composed of three components, as shown in Figure 7. The first component searched the candidate alignment positions for a sequence from the database using the indexes. The second component calculated local alignments around the candidate positions using the Smith-Waterman algorithm [25] for calculating the alignment scores. Finally, the third component sorted the alignment scores and output the search results.

### Construction of Database Indexes

Before searching a database, the indexes for all of the database sequences were constructed. All of the sequences in the database were connected to inserting delimiters to transform them into several long sequences. Index keys were generated for every offset of a *K*-mer in a database sequence. The position at which each key appeared was stored in the order in which it appeared in the database. For large database, the sequences in the databases were divided into several chunks because of the limitation of memory space. In a search process, the system searches for homologues for each database chunk by switching them and then merges the search results. GHOSTM automatically divides a database into chunks according to the upper limit of the database chunk size specified by the user.

### Search for Candidate Alignment Positions

The DNA query sequences were initially translated into protein sequences in all of the six open reading frames. The DNA sequences were then divided into three-letter codons, and each codon was translated into an amino acid according to the genetic code table. There are 3 possible reading frames in a DNA strand, and double-stranded DNA has six different reading frames. If a codon contained an “N”, which means any nucleotide, it was translated into “X”, which means any amino acid.

The index keys of protein sequences were generated in the same way as the database indexes but with *s* character skips. These skips reduce the calculation cost at the expense of search sensitivity in the candidate search component. For confirming matches, a database sequence was first divided into regions of size *r*, and the key of each query was compared with the keys of the database sequences. If more than a threshold number *t* of keys matched in a region and the right adjacent region, the position was stored as a candidate alignment. Figure 8 shows an example of a search result in which three candidate positions were reported with a threshold of *t = 2*.

### Local Alignment

After searching for alignment positions, optimal local alignment was performed for the region around each candidate position using the Smith-Waterman algorithm, and the alignment score for each candidate position was calculated. When calculating the local alignment, we restricted the alignment target of a database sequence to a small region of size *m+2r+2e*, where *m* was the length of the query and *e* was the extension width of an alignment region, as illustrated in Figure 9.

### Mapping to GPUs

Both the candidate search and local alignment components required a large amount of computing time. Therefore, we processed queries on both components in parallel and mapped them onto GPUs. Thus, multiple queries were simultaneously processed on different GPU cores. We used NVIDIA CUDA ver. 2.2 to implement the GPU computing and mapped the two different calculation components as the two kernel functions.

The GPU computing program has several limitations, even with the use of current GPUs and CUDA. Thus, we introduced some techniques to our implementation. First, because it was impossible to access the host memory during GPU execution, the calculation results had to be stored to memory on a GPU. However, the size of the memory on a GPU is limited, and the global memory, which is the largest on a GPU, is also used for storing query sequences, database sequences and indexes. Furthermore, we could not know, a priori, the number of candidates and the size of the results to be stored when we generated a candidate for a large number of queries. Consequently, the storage of the results often failed because of the shortage of GPU memory. To overcome this problem, we first counted the number of candidates at the alignment position and then divided the queries into subqueries, whose results could be stored in the global memory of the GPU.

For the implementation of local alignment, a GPU-accelerated Smith-Waterman algorithm has already been proposed [18], [19]. However, this implementation was designed for alignments between long sequences and required the synchronization of multiple threads. Shorter sequences require more frequent synchronizations, which slows the calculation. Thus, in our proposed system, a thread was assigned to each candidate alignment position, and the synchronization among threads was removed. In the alignment process, all of the threads randomly and frequently accessed the scoring matrix. Thus, the matrix data were stored on the texture memory of a GPU because the access speed was much faster than the global memory of a GPU.

To utilize GPUs with CUDA, we must decide the number of grids, blocks, and threads. We fixed the number of grids, blocks, and threads to 1, 128, and 256, respectively. We optimized these parameters for the Tesla S1070, which we used. These parameters do not affect the performance significantly, but they should be optimized for other types of GPUs to achieve maximum performances.

### Availability and Future Directions

GHOSTM was implemented in C++ and the NVIDIA CUDA library and requires CUDA 2.2 or higher. It is distributed under the MIT license and is available for download at http://code.google.com/p/ghostm/.
